# Spontaneous degradation of methylene blue adsorbed on magnetic biochars

**DOI:** 10.1038/s41598-023-39976-9

**Published:** 2023-09-07

**Authors:** Anton Zubrik, Dávid Jáger, Eva Mačingová, Marek Matik, Slavomír Hredzák

**Affiliations:** Institute of Geotechnics of the Slovak Academy of Sciences, Watsonova 45, 04001 Kosice, Slovakia

**Keywords:** Porous materials, Mass spectrometry, Pollution remediation

## Abstract

Methylene blue (MB) is one of the most widely studied organic molecules in the treatment of wastewater. Sorption, biodegradation, photodegradation, electrochemical oxidation, ozonation, and other advanced oxidative processes are frequently used to remove this dye from water solutions. The unexpected degradation of MB adsorbed on magnetic biochar from aqueous solution was observed. We found that the conditions of handling, such as drying temperature and storage period, substantially influenced the stability of the dye fixed on the (magnetic) carbon adsorbents. Twelve substances were identified by mass spectrometry as products of decomposition, mostly demethylated, oxidated, and hydroxylated substances. The decomposition of MB was further investigated using non-magnetic carbon biochars and aluminosilicate mineral zeolite. Our findings of the spontaneous decomposition of MB and the identification of the species of degradation offer a new approach to evaluate the mechanism of adsorption, the process of regeneration, and the toxicity of treated solutions.

## Introduction

Methylene blue (MB, C_16_H_18_N_3_SCl) is a synthetic basic dye developed by the German chemist Heinrich Caro in the nineteenth century. It belongs to the benzothiazine class of heterocycles. MB is a cationic organic chloride salt having 3,7-bis(dimethylamino)phenothiazin-5-ium as the counterion^1^. This compound is used in biology (staining), chemistry (redox indicator, sulfide analysis), medicine (treatment of methemoglobinemia), and the textile industry^2^. Synthetic dyes are harmful to the environment and ultimately human health, so their effective removal from industrial waterways is an important topic for many academics worldwide. Many procedures have been developed to remove toxic dyes from soil and aquatic environments. According to Santoso et al.^3^ and data collected from 2008 to 2019, the conventional method of adsorption is still one of the most widely investigated methods for removing MB (the number of publications investigating adsorption increased more than twofold within 10 years). Interestingly, when new types of adsorbents are synthesized, MB is frequently chosen as a model molecule to monitor and evaluate the adsorption capacity^4,5^. Much attention has recently focused on the preparation of low-cost adsorbents from local materials such as biomass waste, zeolites, coal, and by-products (carbon chars after pyrolysis). A simple modification or activation (chemical or physical) of these materials can lead to better properties of adsorption involving surface enhancement and pore formation^6,7^. Adsorbents modified with magnetic nanoparticles are easily separated from water solutions using a magnet^8^. The adsorbents can then be regenerated and reused several times.

In addition to the conventional method of adsorption, biodegradation and advanced oxidation processes (e.g. photodegradation, electrochemical oxidation, ozonation, and the Fenton process) have been tested to remove MB from model solutions. During advanced oxidation processes, radicals are produced that can attack specific sites of organic molecules. This process is associated with the mineralization of organic pollutants and the formation of by-products. The main goal is not only color disappearance during the treatment of organic dyes, but also the complete mineralization or conversion of persistent organic pollutants into biodegradable compounds, a combination of advanced oxidation processes with biological treatment. Ramírez-Aparicio et al.^9^ studied the surface photocatalytic degradation of MB on carbon nanostructures. de Oliveira Guidolin et al.^10^ identified the photocatalytic pathway of the degradation of MB in aqueous solutions using magnetite nanoparticles. An enhanced photocatalytic activity was monitored during the degradation of MB using Ag@TiO_2_/WO_3_, a novel nanocomposite^11^. Microwave (MW) plasma jets were applied for degrading MB in water^12^, where the degradation was associated with the formation of H_2_O_2_. Vieira et al.^13^ studied the ecotoxicity of a MB dye solution after degradation by the Fenton process and found that the by-products of degradation were more toxic than the untreated dye. This negative effect was associated with the formation of hydroxyl radicals from the residual H_2_O_2_ and iron.

Our study monitored the unexpected degradation of MB adsorbed on magnetic biochar (MWchar-Mag). MB was first adsorbed on MWchar-Mag in an aqueous solution. After solid/liquid separation, we analyzed the supernatant using UV/VIS spectroscopy for studying the efficiency of adsorption. The sediment (MWchar-Mag/MB) was dried at various temperatures and stored for various periods, and MB was then extracted. The extracts were analyzed by UV/VIS spectroscopy, high-performance liquid chromatography with a diode array detector (HPLC/DAD), and high-resolution mass spectrometry (MS) to monitor mineralization and identify the products of decomposition. The degree of MB degradation was also compared with non-magnetic adsorbents (aluminosilicate mineral zeolite and two types of carbon chars).

## Materials and methods

We made non-magnetic CPchar sample by pyrolyzing wheat (*Triticum aestivum*) straw in a horizontal quartz tube (T = 550 °C, heating rate = 5 °C/min, holding time = 90 min, N_2_ atmosphere). The non-magnetic MWchar sample was produced using microwave-assisted pyrolysis as follows. We combined 0.75 g of CPchar with 14.25 g of raw biomass (wheat straw). CPchar here served as a susceptor and catalyst for microwave heating. We added 15 g of the substance to a quartz flask before adding gaseous nitrogen. The flask was closed, and the contents were pyrolyzed in a Panasonic NN-GD566M microwave oven for 6 min at a frequency of 2.45 Ghz and 900 W.

Magnetic biochar was produced by mixing MWchar with maghemite nanoparticles at a ratio of 1:1 (w/w) following Zubrik et al.^7^. In brief, MWchar was mixed for 60 min with iron ions (Fe^3+^/Fe^2+^ at a 2:1 molar ratio) in water (degassed with N_2_, pH = 2.0). The solution was then treated with 24% ammonium hydroxide. The sample (MWchar-Mag) was allowed to precipitate for 60 min, washed with deionized water to a neutral pH, filtered, and dehydrated at 80 °C.

MB hydrate (purity > 95%, MW = 319.86 g/mol) was purchased from Mikrochem s.r.o. (Pezinok, Slovakia) for investigating the removal of MB using MWchar-Mag and non-magnetic control adsorbents (MWchar, CPchar, and zeolite (Zeomineral Entero, Mád, Hungary)) under the following conditions: batch-type system, room temperature, 24 h adsorption equilibrium time, concentration of adsorbent = 10 g/L, initial concentration (c_0_) of MB = 500 mg/L, equilibrium pH = 10.2. The adsorption tests were carried out in a rotating shaker set to 30 rpm.

After adsorption, the magnetic and non-magnetic samples were removed from the solution using a magnet (for the magnetic adsorbent) or centrifugation (for the non-magnetic adsorbents). The dye from the adsorbent/MB complex was also extracted. The wet adsorbents were first dried at 80 °C and then stored in a desiccator. The drying temperatures and storage periods influenced the stability of the MB fixed on the magnetic biochar. The adsorbent/MB complex was consequently dried at room temperature (RT) under gaseous nitrogen. The effects of drying temperature (40–100 °C) and storage period (from one day to six months) on MB degradation were investigated in the following experiments. In a typical procedure of desorption, 1 ml of methanol was used to extract 20 mg of sample (adsorbent/MB) three times over the course of 30 min. The solid and liquid phases were separated (either magnetically or by centrifugation), and three supernatants were collected, coupled together, and evaporated under gaseous nitrogen at room temperature. The samples were dissolved in deionized water and then examined using HPLC/DAD and UV/VIS spectroscopy. Unmodified carbon chars and zeolite were also compared as control adsorbents of MB.

The efficiency of adsorption was evaluated using the MB concentration in the supernatant. A Helios Gamma UV/VIS spectrophotometer (Thermo Electron Corporation, UK) was used to measure the concentrations of MB. The maximum wavelength for measuring MB was 663 nm. The concentrations were calculated using calibration curves ranging from 0 to 20 mg/L.

A DIONEX UltiMate 3000 HPLC system (Thermo Scientific, Waltham, USA) with a diode array detector was used for the HPLC analysis of MB and its residues. The compounds were separated on a Thermo Scientific Acclaim 120 C18 column (2.1 × 100 mm, 3 µm, 120 Å) with an eluent mixture of 0.1% formic acid in water (A) and 0.1% formic acid in methanol (B). The multistep gradient elution was configured as: 0–1 min, 25% B; 1–4 min, 25–60% B; 4–6 min, 60% B; 6–12 min, 60–80% B; and 12–16 min, 80% B. In isocratic elution mode, 25% B was used for 8 min to stabilize the system before the next injection. The flow rate was set to 0.25 ml/min, the sample injection volume was set to 10 µl, and the column temperature was set to 40 °C. The samples were filtered using 0.22-µm PTFE syringe filters. The HPLC/DAD chromatograms were obtained at 290 and 663 nm. Chromeleon 7.1 (Thermo Scientific, Germany) was used for collecting and processing the data. The HPLC eluate was subsequently examined using an MS micrOTOF-Q II quadrupole-time of flight hybrid mass spectrometer (Bruker Daltonics, Billerica, USA) equipped with an Apollo II ESI electrospray ion source (Bruker Daltonics) operated in positive mode. We used the accurate masses of the molecular and fragment ions from the MS/MS experiments to determine the empirical formulas and to suggest possible species structures. The mass deviations (experimental vs theoretical) for each m/z were < 5 ppm. The correction for retention-time offset between the HPLC/DAD and HPLC/ESI–MS chromatograms was 4 s.

Atomic absorption spectroscopy (AAS; Varian 240 RS/240 Z, Mulgrave, Australia) was used to determine the quantities of metal (Fe) in the solutions and Aqua Regia was used to dissolve the biochar samples. The ash content was determined by burning of sample in a muffle oven at 815 °C to a constant weight. A D8 Advance diffractometer (Bruker AXS, Karlsruhe, Germany) was used to study X-ray powder diffraction (XRD) using Cu Kα_1_ radiation (voltage, 40 kV; current, 40 mA; goniometer step, 0.04°/s; time step, 25 s). A Kappabridge KLY-2 apparatus (Geophysics, Brno, Czech Republic) was used to measure volumetric magnetic susceptibility (κ) under the following conditions: magnetic field intensity of 300 A/m, field homogeneity of 0.2%, and frequency of 920 Hz. The particle morphology was studied by field emission scanning electron microscope, using a TESCAN MIRA3 FE (TESCAN, Czech Republic) with energy dispersive X-ray (EDX) microanalysis system (Oxford Instruments, Abingdon, UK).

## Results

### Characterization of the samples used for MB adsorption

**Figure 1:**
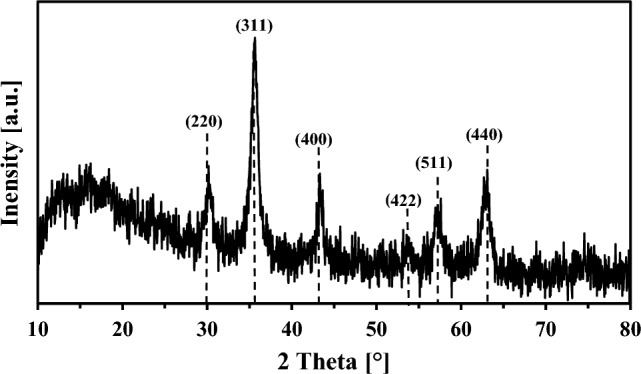
X-ray powder diffraction of magnetic biochar (MWchar-Mag) with Miller indices (h, k, and l) for maghemite.

The findings of the elemental analysis of the magnetic biochar and its precursors (wheat straw biomass, CPchar, and MWchar) are presented in Table 1. Both CPchar and MWchar had much more carbon (58.1% and 58.9%, respectively) than the native biomass (43.1%). The amount of carbon decreased to 28.7% after the modification by iron oxide nanoparticles, and the amount of total iron varied by about 30.3% (± 1.9%). The iron content in unmodified carbon biochars was less than 0.3%. The other elements such as Si, Ca, K, Mg, P were detected by EDX analysis (Figure S1, Supplementary material). The synthesized composite (MWchar-Mag) had a high magnetic susceptibility of 640,074.10^−6^ SI units. Both a Mössbauer spectroscopic investigation^7^ and our XRD examination of MWchar-Mag (Fig. 1) supported the existence of a non-stoichiometric nano-maghemite (γ-Fe_2_O_3_) phase loaded on the carbon matrix.

**Table 1:** Elemental (CHNS) analysis, ash content, and the efficiency of adsorption of the non-magnetic adsorbents and magnetic biochar (MWchar-Mag).

Sample	A^d^ (%)	C^d^ (%)	H^d^ (%)	N^d^ (%)	S^d^ (%)	O^d^ (%)	E (%)
Wheat straw	7.5	43.1	6.1	0.6	0.5	45,7	–
CPchar	28.3	58.1	2.4	1.2	0.4	9.6	30
MWchar	31.5	58.9	1.2	1.0	0.3	7.1	30
MWchar-Mag	36.7	28.7	1.0	0.5	0.1	33.0	49
Zeolite	–	–	–	–	–	–	99

A, ash; d, dry basis; O^d^, difference based on 100 – (A^d^ + C^d^ + H^d^ + N^d^ + S^d^); E, efficiency of removal (batch-type system; room temperature; 24 h; concentration (adsorbent) = 10 g/L; c_0_ (MB) = 500 mg/L; pH = 10.2).

### Adsorption/desorption experiments and degradation of MB

UV/VIS spectrophotometry was used to investigate the efficiency of removal of the three carbon adsorbents (CPchar, MWchar, and MWchar-Mag) and a zeolite sample (Table 1). The zeolite sample had the highest efficiency (99%), with an adsorption capacity of 49 mg of MB per gram under the defined conditions. The efficiency of adsorption was lower in the samples of the CPchar, MWchar and MWchar-Mag. Our investigation of adsorption focused primarily on magnetic biochar.

**Figure 2:**
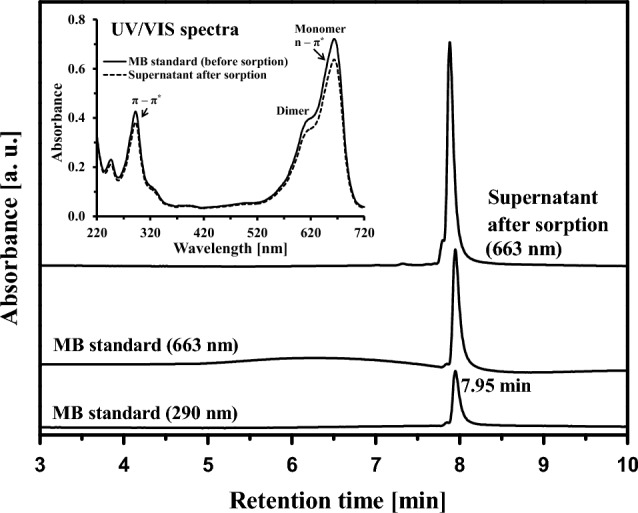
HPLC/DAD profile of the methylene blue standard and HPLC analysis of the magnetically filtered supernatant after adsorption of MB on MWchar-Mag.

The HPLC examination of the MB standard indicated a single peak at 7.95 min (Fig. 2). The supernatant sample contained no additional compounds. Figure 2 (top-left inset) shows the UV/VIS absorbance spectra of the MB standard and the supernatant. The first absorbance maximum was in the UV region at λ_max_ = 290 nm. The other two maxima were in the VIS region (a monomer, λ_max_ = 663 nm; a dimer, λ_max_ = 612 nm). The UV/VIS spectrum of the supernatant after adsorption did not change relative to the standard.

**Figure 3:**
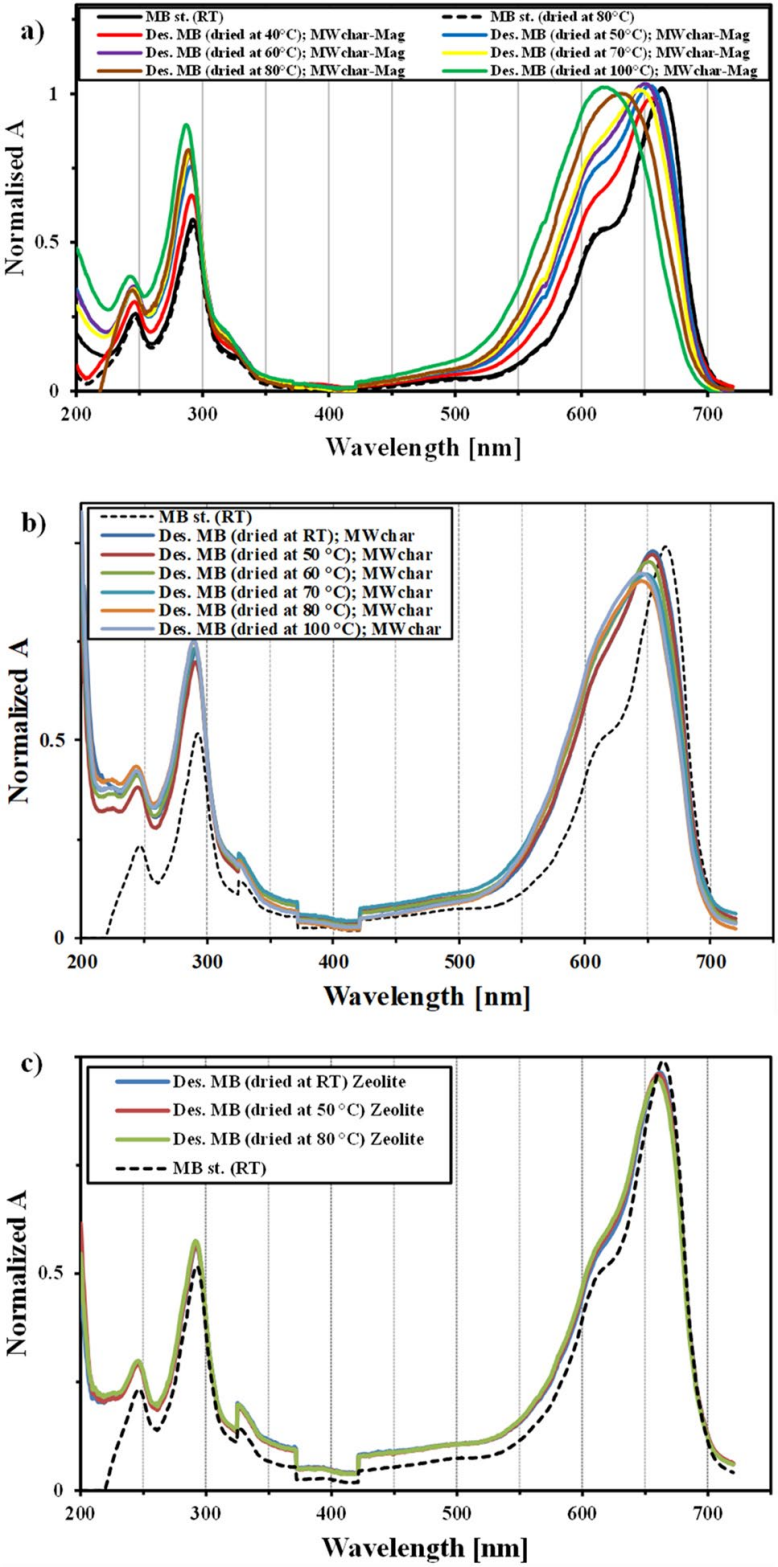
Effect of drying temperature on the UV/VIS profile of dye extracted from magnetic biochar (**a**), MWchar (**b**) and Zeolite (**c**). Note: the sorbents were stored for seven days in a desiccator after drying at a defined temperature. The dye was then desorbed using methanol (Abbreviations: Des, desorption; RT, room temperature).

After adsorption, the MWchar-Mag/MB sample was dried and stored in a desiccator for testing desorption. Methanol was used to remove the dye from the magnetic biochar. The methanol was evaporated, and the extract was dissolved in water and then analyzed by UV/VIS spectroscopy (Fig. 3a). The UV/VIS profile of the extract from the sample MWchar-Mag/MB dried at higher temperatures (60–100 °C) differed from the MB standards. Maximum wavelengths in either the VIS or UV region shifted to lower wavelengths, two absorbance peaks, at 612 and 663 nm, ultimately disappeared and new maxima were observed (see the UV/VIS spectra at different drying temperatures). The degradation of the MB molecule on the magnetic biochar was not expected, because the MB spectrum of the supernatant after adsorption in the alkaline solution did not change in either the UV or VIS region. In comparison with magnetic biochar, the UV/VIS profile of MWchar/MB evolved differently (Fig. 3b). The UV/VIS spectrum minimally modified in the case of zeolite samples (Fig. 3c).

Our objectives were to determine why the UV/VIS profile of the extracted dye changed and whether the MB molecule fixed on the adsorbents remained intact or if it degraded during drying and/or storage. We used HPLC with DAD and MS detection instead of UV/VIS spectroscopy to explore this phenomenon.

**Figure 4:**
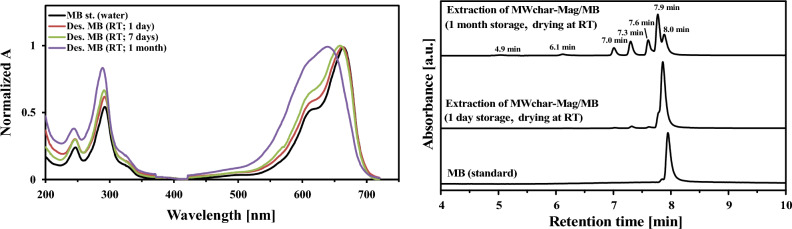
Effect of storage period on the UV/VIS (left) and HPLC/DAD (right) profiles of dye extracted from MWchar-Mag (Abbreviations: Des, desorption; RT, room temperature).

The effect of storage period when the samples were dried at room temperature (under gaseous nitrogen) was examined. Figure 4 (left) shows the spectra of the UV/VIS profile, and Fig. 4 (right) shows the HPLC/DAD analysis. The UV/VIS spectra and the HPLC profile changed negligibly after one day of storage. Both the UV/VIS and HPLC/DAD measurements differed substantially after one month of storage.

The next experiments focused on the identification of the degradation products and their percent distributions. The MWchar-Mag/MB extracts were analyzed using two methods, HPLC/DAD and HPLC/ESI–MS, for identifying the degradation products and determining their relative distributions. The MS spectra with molecular ions were further fragmented to obtain MS/MS spectra. The HPLC/MS analyses found that MB spontaneously degraded to substances with lower molecular weights. We identified 12 substances using the accurate monoisotopic masses of the protonated molecular ions and fragment ions from the MS/MS experiments. The suggested formulas, names, and structures of the products identified are listed in Table 2.

**Table 2:** Compounds identified from the HPLC/ESI–MS/MS analysis.

Retention time [min]	m/z of protonated molecular ion	Proposed formula/name	Proposed structure
**HPLC/ESI–MS**			
6.1	228.0590	C_12_H_10_N_3_S^+^ (Thionine)	
7.0	242.0751	C_13_H_12_N_3_S^+^ (Azure C)	
7.0	229.0428	C_12_H_9_N_2_OS^+^	
7.3	243.0594	C_13_H_11_N_2_OS^+^	
7.3	256.0908	C_14_H_14_N_3_S^+^ (Azure A)	
7.6	256.0908	C_14_H_14_N_3_S^+^	
7.9	257.0750	C_14_H_13_N_2_OS^+^	
7.9	270.1064	C_15_H_16_N_3_S^+^ (Azure B)	
8.0	284.1222	C_16_H_18_N_3_S^+^ (Methylene blue)	
**Species identified by MS ion extraction**			
3.2	284.0485	C_14_H_10_N_3_O_2_S^+^	
5.0	256.0539	C_13_H_10_N_3_OS^+^	
5.3	302.0594	C_14_H_12_N_3_O_3_S^+^	
9.5	230.0270	C_12_H_8_NO_2_S^+^	

**Figure 5:**
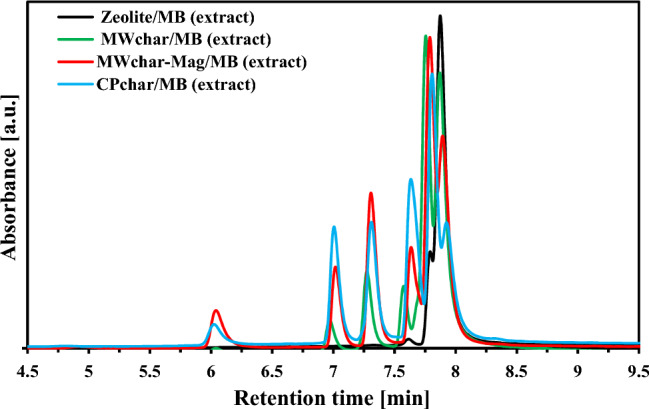
HPLC/DAD analysis of the extracts after the adsorption of MB on the samples of magnetic biochar, MWchar, and CPchar. The samples were dried at 80 °C and stored for one day in a desiccator.

Table 3 presents details of the effects of storage period (sample MWchar-Mag) and temperature (MWchar-Mag; MWchar and Zeolite sample) on MB degradation. The effect of the storage period on MWchar-Mag/MB was studied by drying the sample at 80 °C. The negative impact of time was monitored. MB was almost completely degraded (the remaining 1.8%) when the sample was dried at 80 °C and stored for six months. We recorded the percent increase in the amounts of compound at retention times of 6.1 and 7.0 min. The combination of one month of storage and a drying temperature of 100 °C decreased the MB concentration by 8.6% in the case of MWchar-Mag. Another aim of our study was to compare the results from the adsorption/desorption of the magnetic biochar with the results for the non-magnetic adsorbents (MWchar and zeolite). The process of adsorption and extraction were the same as for MWchar-Mag. When MB was adsorbed on unmodified carbon adsorbent (MWchar) (Table 3; Fig. 5), it also degraded. In in comparison to the magnetically modified adsorbent, the results reveal a lower degree of degradation. The lowest effect was observed with inorganic zeolite adsorbent. The number of degradation products and their concentrations generally increased after a longer storage period and higher drying temperatures. Overall, the degree of degradation was highest with magnetic adsorbent (MWchar-Mag).

**Table 3:** Distribution of compounds identified from the HPLC relative area (DAD, 290 nm): the effects of storage period and drying temperature.

Retention time [min]	4.9	6.1	7.0	7.3	7.6	7.8	7.9
***Monoisotopic mass (m/z ion)***	no MS	228.059	229.044	243.060	256.090	257.075	284.122
	signal		242.075	256.091		270.106	
	Distribution [%]						
**MWchar-Mag**							
***Effect of storage period (drying at 80 °C)***							
1 day storage	0.0	2.3	3.2	7.8	4.5	29.7	52.5
7 days storage	0.4	4.0	5.2	10.5	7.3	33.3	39.4
1 month storage	0.9	7.7	13.6	19.2	15.4	27.3	15.9
6 months storage	3.5	28.3	29.1	13.1	13.1	11.1	1.8
***Effect of drying temperature (1 day storage)***							
RT	0.3	2.3	1.8	4.7	1.3	15.5	74.1
50 °C	0.3	3.1	2.4	6.1	2.4	19.5	66.3
60 °C	0.0	2.2	2.8	5.6	3.9	24.1	61.5
70 °C	0.5	3.7	2.9	6.8	2.7	20.9	62.6
80 °C	0.0	2.3	3.2	7.8	4.5	29.7	52.5
100 °C	0.0	3.8	7.1	13.9	9.7	35.7	29.8
***Effect of drying temperature (7 days storage)***							
RT	0.7	2.6	2.6	6.2	3.4	25.3	59.3
50 °C	0.6	4.1	3.9	8.0	4.9	27.5	51.1
60 °C	0.3	3.0	3.8	7.4	5.8	30.9	48.7
70 °C	1.1	4.6	4.3	8.5	4.8	28.2	48.5
80 °C	0.4	4.0	5.2	10.5	7.3	33.3	39.4
100 °C	0.3	5.0	8.7	14.7	11.1	34.9	25.2
***Effect of drying temperature (1 month storage)***							
50 °C	1.0	3.2	7.5	14.0	14.0	33.8	26.4
60 °C	0.8	3.7	9.1	15.4	15.2	32.4	23.4
70 °C	0.7	4.0	10.9	16.8	16.0	31.3	20.4
80 °C	0.9	7.7	13.6	19.2	15.4	27.3	15.9
100 °C	0.8	9.1	21.9	23.3	16.1	20.1	8.6
**MWchar**							
***Effect of drying temperature (1 day storage)***							
RT	0.0	0.4	1.2	3.0	2.1	22.2	71.1
50 °C	0.0	0.3	1.1	2.6	2.2	24.0	69.8
60 °C	0.0	0.7	2.1	4.3	3.3	28.5	61.1
70 °C	0.0	0.7	2.4	6.0	4.4	32.3	54.3
80 °C	0.0	1.0	3.6	9.1	6.0	37.1	43.2
100 °C	0.0	1.0	3.9	8.2	7.1	38.9	41.0
***Effect of drying temperature (7 days storage)***							
RT	0.0	0.7	2.4	5.7	4.1	31.0	56.3
50 °C	0.0	0.9	2.8	5.3	5.6	33.6	51.9
60 °C	0.4	1.4	3.9	7.6	6.2	34.5	46.0
70 °C	0.4	1.6	4.5	8.9	6.3	35.6	42.8
80 °C	0.0	2.8	5.7	11.7	7.4	35.8	36.7
100 °C	0.0	3.0	5.8	9.6	8.0	37.6	36.1
**Zeolite**							
***Effect of drying temperature (1 day storage)***							
RT	0.0	0.0	0.1	0.5	1.1	14.0	84.3
50 °C	0.0	0.0	0.2	0.5	1.3	14.8	83.3
80 °C	0.0	0.0	0.2	1.2	1.6	18.5	78.6
***Effect of drying temperature (7 days storage)***							
RT	0.0	0.0	0.1	0.5	1.0	15.4	83.0
50 °C	0.0	0.0	0.2	0.8	1.4	18.5	79.2
80 °C	0.0	0.0	0.4	1.5	2.0	20.6	75.5

Note that the UV/VIS absorbance (Figure S3, Supplementary material) showed that the absorbance profiles of all supernatants were identical to the MB standard (the stability of MB heated to 100 °C was also confirmed). On the other hand, the results of the analysis of the adsorbent/MB extracts differed. Figure 5 presents HPLC chromatograms of MB adsorbed of unmodified carbon chars and zeolite. In the case of Zeolite, partial MB decomposition was detected as well. The HPLC/ESI–MS analysis of both extracts (CPchar/MB and MWchar/MB) and MWchar-Mag/MB identified the same degradation products.

## Discussion

MB is a cationic organic chloride salt with the cation 3,7-bis(dimethylamino)phenothiazin-5-ium. The usual ionic form (MB^+^) can create sandwich-type H-aggregates (dimers and trimers) in water. MB is a synthetic basic dye with sensitive oxidation/reduction properties. The light absorption of MB depends on its concentration, type of solvent, and adsorption to specific materials and other interactions. The monomeric (MB^+^) form is represented in aqueous environments by an absorption band between 650 and 675 nm. Lower wavelengths (600–625 nm) localized the dimeric form (MB_2_^2+^) (Fig. 2, inset). The absorbance peak at 663 nm corresponds to n – π^*^ transitions, and π – π^*^ transitions are responsible for absorbance near 290 nm in the UV region^14^.

The decomposition of MB dye fixed on magnetic biochar and non-magnetic adsorbents was confirmed in this study. The dye nevertheless degraded, even though no external irradiation associated with the production of radicals was applied. Drying temperature and storage period strongly influenced MB mineralization. Two processes, from our point of view, can contribute to the spontaneous degradation of MB. The first process is associated with self-aggregation during adsorption, and the second process is the formation of active sites/functional groups on the adsorbents, which can influence the stability of the adsorbed compound.

MB dye has complicated physical and chemical properties. It has several resonant structures, including a monomer, a dimer, an H-aggregate, and an MB hydrate^15^. The dominant form is affected by the ambient environment (e.g. type of solvent, concentration). The self-association to higher oligomeric forms can thus be ensured when MB^+^ interacts with solid surfaces during sorption. Lakshminarasimhan et al.^16^ reported an MB metachromasy due to aggregation over phosphate-modified polymeric carbon nitride. A dimer and trimer were formed due to MB self-aggregation. Fernández-Pérez and Marbán^17^ investigated MB self-aggregation in water at various concentrations and temperatures. Contrary to the common belief that the trimer is the dominant aggregate at high MB concentrations, Fernández-Pérez and Marbán^17^ found that the tetramer acted alone, without any counterion at high MB concentrations.

We assumed that in addition to self-aggregation, oxidation/reduction reactions and demethylation must have been involved in the adsorption of MB on the surface of the adsorbent. Ovchinnikov et al.^15^ proposed that protolytic substances such Azure A, Azure B, Azure C, thionine, thionoline, thionol, methyl thionolin, and others could be produced under oxidation–reduction conditions. Our experiments confirmed this theory: HPLC/MS analyses indicated that MB could degrade into substances with low molecular weights (Table 2). A nucleophilic attack of OH^−^ ions on MB in alkaline media was observed^18^. Theoretical studies and visualization of the electronic structures (HOMO and LUMO) of MB indicated that the sulphur atom in the heterocycle ring and two carbon atoms (positions 3, 7) in benzene rings are the preferred sites for nucleophilic attacks. We presumed a similar effect, where the molecule sequentially loses chromophores by the oxidation, hydroxylation, hydrolysis, and cleavage of the bonds in the functional groups by the action of reactive oxygen species catalytically generated from water on the vicinity/surface of the adsorbents. The aromatic system remained intact during the transformation; HPLC–MS found no open-ringed products.

Organic pollutants are generally frequently degraded by the application of advanced oxidation processes (e.g. photo-Fenton, Fenton-like, and Fenton oxidation) involved in the generation of radicals. The pollutant molecules are cleaved into substances with lower molecular weights by reactions with hydroxyl radicals (·OH). da Silva et al.^19^ used a combination of adsorption and a Fenton reaction to remove MB dye from model wastewater. The Fenton oxidation reaction was initiated by the external addition of H_2_O_2_. Both pure activated carbon (from peanut hulls) and iron-based catalysts were able to remove MB by simultaneous adsorption and an oxidation reaction. Smaller crystals of magnetite and the presence of Fe^2+^ ions can increase catalytic activity in connection with the generation of surface oxygenated groups, thereby increasing the efficiency of MB removal from the aquatic environment. We did not use external irradiation connected with the formation of radicals, with only the storage conditions affecting the degradation of MB. The presence of radical reaction in our study is therefore questionable.

## Conclusions

The degradation of MB adsorbed on magnetic and non-magnetic biochars in aqueous solution was unexpected. Drying and storage conditions had strong impacts on the stability of the dye fixed on the (magnetic) carbon adsorbents. MB transformed/degraded into substances with lower molecular weights after adsorption/desorption. We used high-performance liquid chromatography and electrospray mass spectrometry to identify twelve compounds, predominantly demethylated, oxidized, and hydroxylated compounds, and proposed their structures. The synthesized (magnetic) biochars had catalytic activity during the degradation of MB. We assume that the degradation of the dye was mediated by nucleophilic attack of the oxygen species on the surface of the (magnetic) biochar.

### Supplementary Information

Supplementary Information.

## Data Availability

The datasets generated and/or analysed during the current study are available in the ZUBMBdata repository, https://zenodo.org/record/7970002#.ZG80U3ZBzql.
