# Supplementary material for: Spontaneous degradation of methylene blue adsorbed on magnetic biochars
Source: Sci Rep. 2023 Sep 7;13:14773. doi: 10.1038/s41598-023-39976-9 (PMC10484945; doi:10.1038/s41598-023-39976-9)
Supplement: Supplementary file 1 — Supplementary Information. [file 41598_2023_39976_MOESM1_ESM.docx]

**Supplementary material**

Scientific Repords

**Spontaneous degradation of methylene blue adsorbed on magnetic biochars**

Anton Zubrik^1,*^, Dávid Jáger^1^, Eva Mačingová^1^, Marek Matik^1^, Slavomír Hredzák^1^

^1^Institute of Geotechnics of the Slovak Academy of Sciences, Watsonova 45, SK-04001 Košice, Slovakia

^*^ Corresponding author: Anton Zubrik

Tel.: +421 557922630, Fax: +421 7922604

e-mail address: zubant@saske.sk

Postal address: Institute of Geotechnics, Slovak Academy of Sciences, Watsonova 45, 04001 Košice, Slovakia

**Contents**

| Figure S1 | Page 2 |
| --- | --- |
| Figure S2, Figure S3 | Page 3 |
|  |  |

**Figure S1.** Field emission scanning electron micrographs and EDX analysis of the samples: a) MWchar-Mag; b) ash of MWchar-Mag; c) MWchar; and d) ash of MWchar.

**Figure S2.** Effect of contact time on the removal efficiency of MB (Conditions: pH 10.2; c(adsorbent) = 10g/L; c(MB) = 500 ppm).

**Figure S3.** UV/VIS absorption spectra of the MB standards, supernatants (after adsorption of MB on unmodified carbon chars and zeolite), and their extracts. The samples were dried at 80 °C and stored for one day in a desiccator.
